# Inhibiting P2Y12 in Macrophages Induces Endoplasmic Reticulum Stress and Promotes an Anti-Tumoral Phenotype

**DOI:** 10.3390/ijms21218177

**Published:** 2020-10-31

**Authors:** Nataša Pavlović, Maria Kopsida, Pär Gerwins, Femke Heindryckx

**Affiliations:** 1 Medical Cell Biology, Uppsala University, 75123 Uppsala, Sweden; Natasa.pavlovic@mcb.uu.se (N.P.); Maria.Kopsida@mcb.uu.se (M.K.); Par.Gerwins@mcb.uu.se (P.G.); 2 Radiology, Uppsala University Hospital, 75237 Uppsala, Sweden

**Keywords:** inflammation, purinergic receptors, macrophages, liver disease, cancer

## Abstract

The P2Y12 receptor is an adenosine diphosphate responsive G protein-coupled receptor expressed on the surface of platelets and is the pharmacologic target of several anti-thrombotic agents. In this study, we use liver samples from mice with cirrhosis and hepatocellular carcinoma to show that P2Y12 is expressed by macrophages in the liver. Using in vitro methods, we show that inhibition of P2Y12 with ticagrelor enhances tumor cell phagocytosis by macrophages and induces an anti-tumoral phenotype. Treatment with ticagrelor also increases the expression of several actors of the endoplasmic reticulum (ER) stress pathways, suggesting activation of the unfolded protein response (UPR). Inhibiting the UPR with tauroursodeoxycholic acid (Tudca) diminishes the pro-phagocytotic effect of ticagrelor, thereby indicating that P2Y12 mediates macrophage function through activation of ER stress pathways. This could be relevant in the pathogenesis of chronic liver disease and cancer, as macrophages are considered key players in these inflammation-driven pathologies.

## 1. Introduction

Hepatocellular carcinoma (HCC) is a primary liver cancer that usually develops in a background of inflammation and chronic liver disease. Liver macrophages are phagocytotic immune cells that form the cornerstones in the inflammation-driven pathogenesis of liver cirrhosis and HCC [1]. Due to their location in the sinusoids and their ability to rapidly respond to a seemingly infinite variety of activating signals, they are the primary cells to react in case of liver injury [2]. Macrophages are heterogeneous by nature, as they play an active role in both inducing and resolving the inflammatory response [2,3]. The ability of macrophages to be reprogrammed or repolarized is an active area of research, especially in HCC, where macrophages have been reported to play a dual role depending on their polarization state [4]. Firstly, classically activated macrophages, often called M1 macrophages, exert a pro-inflammatory T helper type 1 (Th1) immune response and induce a tumoricidal activity [5]. Secondly, alternatively activated macrophages, often referred to as M2 macrophages or pro-tumoral macrophages, are associated with the anti-inflammatory T helper 2 (Th2) immune response and exert a pro-tumoral activity [6,7]. The balance between these anti-tumoral (M1) and pro-tumoral (M2) macrophages plays an important role in the development and progression of liver cancer. One of the factors involved in the switch between an anti- and pro-tumoral response of macrophages is the activation of the endoplasmic reticulum (ER) stress pathways. Endoplasmic reticulum stress is a cellular stress response that occurs when unfolded or misfolded proteins accumulate in the lumen of the ER, which leads to the induction of the unfolded protein response (UPR). This is sensed via three ER transmembrane proteins, namely, protein kinase R-like ER kinase (PERK), inositol-requiring enzyme 1 (IRE1α) and activating transcription factor 6 (ATF6), which become activated after dissociation from the ER chaperone binding immunoglobulin protein (BiP) [8]. Actors of the ER stress pathways are involved in numerous inflammatory processes, including the regulation of macrophage function and polarization [9,10,11]. It is therefore not surprising that ER stress pathways are crucial players in the pathogenesis of chronic liver diseases and HCC [11,12,13,14,15].

The P2Y12 receptor is an adenosine diphosphate (ADP) responsive G protein–coupled receptor expressed on the surface of platelets and is the pharmacologic target of several anti-thrombotic agents [16]. While the function of P2Y12 on platelets has been described extensively, less is known about the role of these receptors on other cell types [17]. However, extracellular ADP and related nucleotides promote a wide range of pathophysiological responses via activation of cell surface G-protein coupled purinergic receptors, while almost every cell type expresses P2 receptors. While ADP is predominantly known for its central role in the cellular energy metabolism, it is also widely distributed outside the cell where it influences diverse biological processes, including the inflammatory cell response. Platelets and tumor cells are known to release a great amount of adenosine triphosphate (ATP) and ADP in the tumor microenvironment, which could influence the inflammatory response through activation of P2Y12 [17]. Extracellular ATP has been shown to induce a strong pro-inflammatory response, whereas ADP generally serves as a negative feedback mechanism to promote an anti-inflammatory (M2) response and aid in the resolution of inflammation [18]. Even though not much is known about the role of P2Y12 in inflammatory cells, studies have reported that it is functionally present in microglial cells, the resident immune cells of the brain, where it plays a role in their activation [19,20]. In addition, other studies have shown a high expression of P2Y12 receptors in lymphocytes, monocytes and megakaryocytes [21], as well as cancer cells [22] and vascular smooth muscle cells [23]. However, the role of P2Y12 in these immune cells remains largely unknown. In this study, we show that P2Y12 is expressed by hepatic macrophages and provide evidence that this receptor plays a role in mediating macrophage polarization and function by inducing ER stress. Macrophages are considered key players in inflammatory pathologies, and altering their activation state and function could be relevant for patients with inflammation-driven cirrhosis and HCC. 

## 2. Results

### 2.1. The P2Y12 Receptor Is Expressed in Mouse Models for Liver Cirrhosis and Hepatocellular Carcinoma

**Figure 1:**
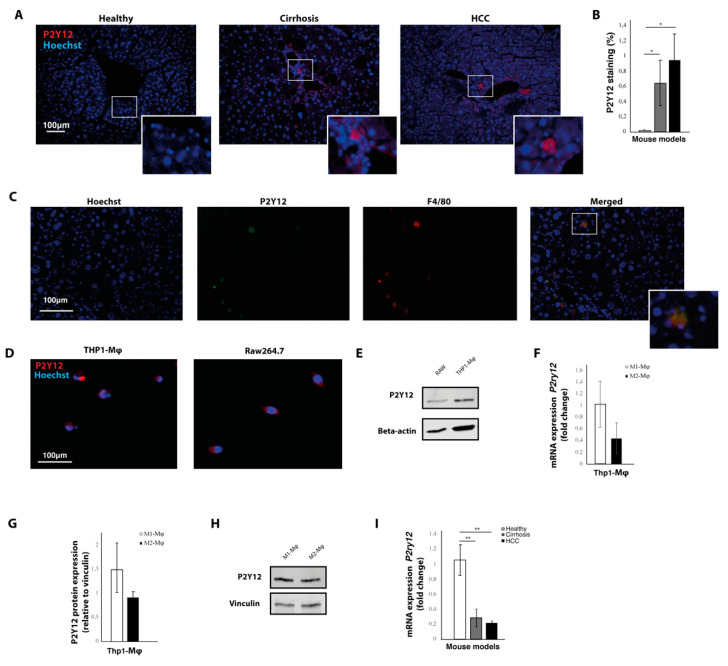
The P2Y12 receptor is expressed in mouse models for liver cirrhosis and hepatocellular carcinoma. (**A**) Immunohistochemical staining with P2Y12 antibodies of liver sections from healthy mice, mice with carbon tetrachloride (CCL_4)_-induced liver cirrhosis and mice with diethylnitrosamine (DEN)-induced liver cancer. (**B**) Quantification of immunohistochemical staining with P2Y12 antibodies. (**C**) Co-staining of P2Y12 and macrophage marker F4/80 in liver slides from DEN-induced hepatocellular carcinoma (HCC). (**D**) Immunocytochemical staining of THP1-differentiated macrophages and Raw264.7 cells. (**E**) Western Blot image of P2Y12 protein expression and beta-actin protein in THP1-differentiated macrophages and Raw264.7 cells. (**F**) mRNA expression levels of *P2yr12* in interferon gamma and lipopolysaccharide-stimulated anti-tumoral (M1) macrophages and interleukin (IL)-13 and IL-4-stimulated pro-tumoral (M2) macrophages (**G**) Quantification of protein expression in pro-tumoral (M2) and anti-tumoral (M1) macrophages. (**H**) Representative image of Western blot showing P2Y12 protein expression in pro-tumoral (M2) and anti-tumoral (M1) macrophages. (**I**) mRNA expression of *P2yr12* in livers from healthy mice, mice with CCL4-induced liver cirrhosis and mice with DEN-induced liver cancer. * = *p* < 0.05; ** = *p* < 0.01.

Liver tissue from healthy mice, mice with carbon tetrachloride (CCL_4_)-induced liver cirrhosis and mice with diethylnitrosamine (DEN)-induced HCC were immunohistochemically stained with P2Y12 antibodies (Figure 1A). This revealed a significant increase in the expression of P2Y12 in cirrhotic and HCC livers (Figure 1B), which was mainly distributed in the peri-vascular areas (Figure 1A). Co-staining of P2Y12 antibodies with F4/80 antibodies showed that P2Y12 expression colocalized with F4/80-positive macrophages (Figure 1C). This was further confirmed in vitro, as THP1-differentiated macrophages and the murine macrophage cell line RAW264.7 expressed the P2Y12 protein, shown by immunohistochemical staining (Figure 1D) and Western blot (Figure 1F). To assess whether P2Y12 levels would differ between pro-tumoral or anti-tumoral macrophages, P2Y12 protein levels were determined in interferon gamma and lipopolysaccharide-stimulated anti-tumoral (M1) macrophages and interleukin (IL)-13 and IL-4-stimulated pro-tumoral (M2) macrophages using Western blot and real-time qPCR (Figure 1F,G). No significant differences were seen in P2Y12 mRNA (Figure 1F) and protein expression (Figure 1G,H) between pro-tumoral (M2) and anti-tumoral (M1) macrophages.

Real-time qPCR was performed to determine the mRNA expression of *P2ry12* in liver tissue from HCC, cirrhotic and healthy mice. In contrast to the results from the quantification of the immunohistochemical staining, we found that the mRNA expression was significantly decreased in the livers from mice with liver cirrhosis and liver cancer, compared to mRNA levels from healthy controls (Figure 1D). This discrepancy between protein levels and mRNA expression of *P2ry12*, has also been observed in other studies [24]. Thrombin stimulation has been shown to induce a modest and transient increase in mRNA expression of *P2ry12* in vascular smooth muscle cells, while protein and cell surface expression of P2Y12 remain markedly increased over prolonged periods of time [24].

### 2.2. Expression of P2Y12 Is Located in the Stroma of Patients with Hepatocellular Carcinoma

**Figure 2:**
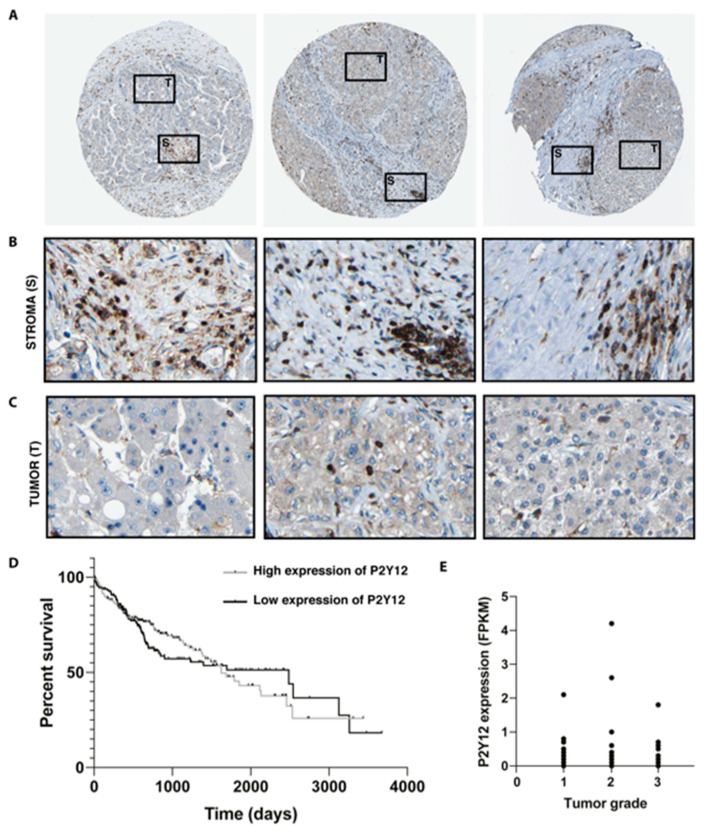
Expression of P2Y12 is located in the stroma of patients with hepatocellular carcinoma. (**A**) Micrographs of hepatocellular carcinoma biopsies stained with P2Y12 antibodies, obtained from the Human Protein Atlas, marking tumor (T) and stomal (S) area [25,26]. (**B**) Detail of stroma and (**C**) tumor areas within the biopsies [26]. (**D**) Kaplan–Meier survival curve of HCC patients with high or low expression of P2Y12 obtained through the Human Protein Atlas [27]. (**E**) P2Y12 expression in different HCC stages, measured as number fragments per kilobase of exon per million reads (RPKM) from the Human Protein Atlas [27].

To assess whether P2Y12 is expressed in patients with liver cancer, micrographs from liver biopsies were obtained from the Human Protein Atlas (Figure 2A) [25,26]. Histopathological evaluation of these liver biopsies stained with P2Y12 antibodies showed that the expression was mainly localized in the peritumoral areas and the fibrotic stroma (Figure 2B). Little to no staining was observed within malignant hepatocytes inside the tumor area (Figure 2C). There was no significant difference (*p* = 0.06) in survival between patients with high or low expression of P2Y12 (Figure 2D). No significant differences of P2Y12 expression were seen between the different tumor grades of HCC, (Figure 2E). Survival data and P2Y12 expression in different stages of HCC was obtained from publicly available data from the Human Protein Atlas [27].

### 2.3. Inhibition of the P2Y12 Receptor Decreases Viability of Macrophages

**Figure 3:**
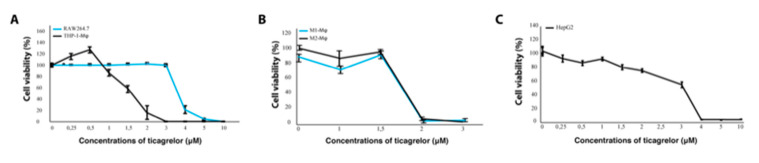
Inhibition of the P2Y12 receptor decreases viability of macrophages. (**A**) Cell viability of THP1-differentiated macrophages and RAW264.7 cells exposed to 0–10 μM ticagrelor measured via a resazurin reduction assay. (**B**) Cell viability THP1-differentiated macrophages primed towards pro-tumoral (M2-φ) and anti-tumoral (M1-φ) macrophage phenotype and exposed to 0–3 μM ticagrelor. (**C**) Viability of HepG2 cells exposed to 0–10 μM ticagrelor.

To study the effect of P2Y12 in vitro, we used the reversibly binding P2Y12 receptor antagonist ticagrelor. To determine if ticagrelor affected cell viability of macrophages and tumor cells, THP1-differentiated macrophages, RAW264.7 and HepG2 cells were exposed to 0–10 μM ticagrelor for 24 h. Inhibition of the P2Y12 receptor with ticagrelor decreased cell viability of both macrophage cell lines in a dose-dependent manner (Figure 3A). The THP1-differntiated macrophages were markedly more sensitive to ticagrelor treatment, as the concentrations that induced a 50% decrease in cell viability (IC-50 values) were 1.7 μM for THP1 macrophages and 4.4 μM for RAW264.7 cells. Since RAW264.7 cells are more differentiated and more heterogeneous by nature, resulting in the possible selection of separate subclones which could affect results [28], THP1-differentiated macrophages were used as a model system for our subsequent experiments [29]. To assess whether ticagrelor would have a different effect in pro-tumoral or anti-tumoral macrophages, the cell number of interferon gamma and lipopolysaccharide-stimulated anti-tumoral (M1) macrophages and IL-13 and IL-4-stimulated pro-tumoral (M2) macrophages was measured via a resazurin reduction assay. No significant differences were seen, thus suggesting that P2Y12 inhibition does not selectively affect survival of a specific macrophage phenotype (Figure 3B). Ticagrelor also decreased tumor cell viability, with a notable decrease in cell viability in concentrations higher than 2 μM (Figure 3C). 

### 2.4. Ticagrelor Enhances Phagocytosis of Tumor Cells

**Figure 4:**
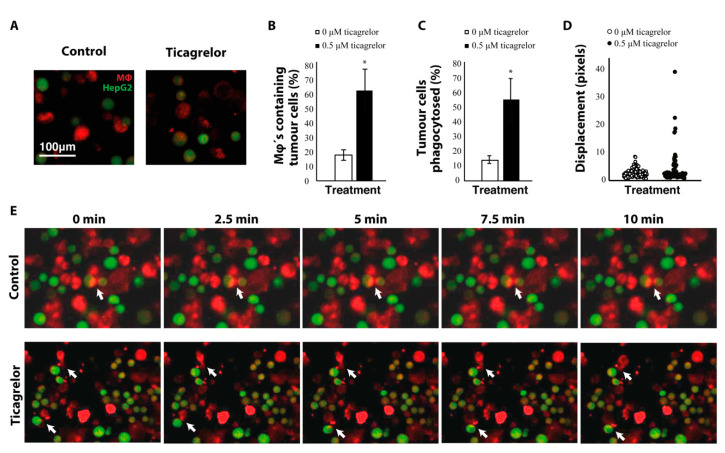
Ticagrelor enhances phagocytosis of tumor cells. (**A**) Confocal microscopy images of macrophages (Mφ) and HepG2 cells stained with CellTracker Red CMTPX and Green CMFDA, respectively. (**B**) quantification of the percentage of macrophages that have phagocytosed tumor cells. (**C**) quantification of the percentage of tumor cells that were removed by macrophages. (**D**) Displacement of macrophages measured on time-lapse videos of the phagocytosis assay. (**E**) time-series of the phagocytosis assay with and without ticagrelor. Arrows represent macrophages actively phagocytosing HepG2-cells * = *p* < 0.05.

To assess whether ticagrelor would affect macrophage function, we looked at phagocytosis of tumor cells by fluorescently labelling macrophages (CellTracker Red CMTPX) and tumor cells (CellTracker Green CMFDA) in a co-culture (Figure 4A,E). Macrophages that phagocytosed tumor cells were then identified by the occurrence of both green and red staining within the same cell. Treatment with ticagrelor lead to a significant increase in the number of macrophages that phagocytosed tumor cells (Figure 4B) and also increased the percentage of tumor cells that were removed by macrophages (Figure 4C). Ticagrelor significantly increased migration of macrophages (Figure 4D), as measured by quantifying displacement of the cells in 10-min time-lapse videos (Figure 4E and Supplementary Materials 1 and 2).

**Figure 5:**
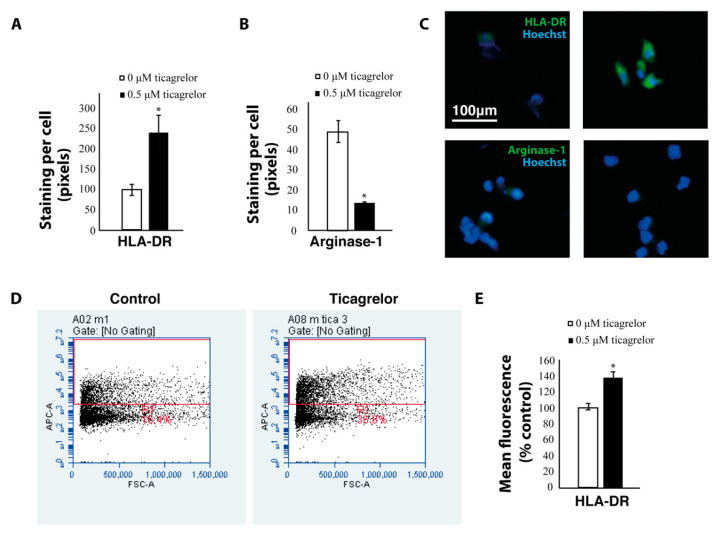
Ticagrelor increases the expression of anti-tumoral markers. (**A**) Area of positive staining with HLA-DR antibodies in THP1 macrophages treated with ticagrelor and controls. (**B**) Area of positive staining with arginase-1 antibodies in THP1 macrophages treated with ticagrelor and controls. (**C**) Representative images of THP1 macrophages stained with HLA-DR and arginase-1 antibodies. (**D**) Flow cytometry analysis of HLA-DR expression in THP1 macrophages treated with ticagrelor (**E**) Quantification of the flow cytometry analyses * = *p* < 0.05.

To see whether this difference in macrophage function was related to their polarization state, we stained macrophages with antibodies against human leukocyte antigen–DR-isotype (HLA-DR) (a pro-inflammatory M1 macrophage marker) and arginase-1 (a pro-tumoral M2 marker). Ticagrelor significantly increased expression of the pro-inflammatory macrophage marker HLA-DR and decreased expression of the pro-tumoral macrophage marker arginase-1 in THP1 macrophages stained with antibodies against HLA-DR and arginase-1, respectively (Figure 5A–C). The increased expression of HLA-DR was also confirmed using flow cytometry (Figure 5D,E). These results indicate that P2Y12 could play a role in mediating macrophage polarization and function.

### 2.5. Expression of ER Stress Genes Is Increased after P2Y12 Inhibition with Ticagrelor

**Figure 6:**
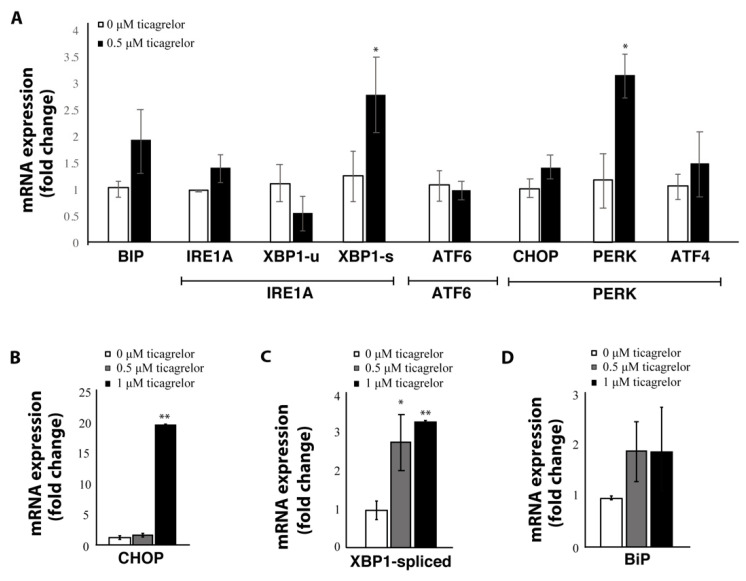
Expression of ER stress genes is increased after P2Y12 inhibition with ticagrelor. (**A**) mRNA expression of different ER stress genes covering the three arms of the unfolded protein response (inositol-requiring enzyme alpha (IRE1α), activating transcription factor 6 (ATF6) and protein kinase R-like endoplasmic reticulum kinase (PERK)) in THP1-differentiated macrophages treated with, 0.5 µM ticagrelor. (**B**) CHOP in THP1-differentiated macrophages treated with 0 µM, 0.5 µM and 1 µM ticagrelor. (**C**) mRNA expression of spliced XBP1 in THP1-differentiated macrophages treated with 0 µM, 0.5 µM and 1 µM. (**D**) mRNA expression of BIP in THP1-differentiated macrophages treated with 0 µM, 0.5 µM and 1 µM ticagrelor. * = *p* < 0.05; ** = *p* < 0.01.

One important mediator of the phenotypic shift between anti-tumoral and pro-tumoral macrophages is the activation of the UPR through the ER stress pathways. To assess whether actors of the ER stress pathways were affected by P2Y12 inhibition, we measured mRNA expression of markers from the three arms of the UPR, namely IRE1α, ATF6 and PERK. The most profound effect was through actors of the PERK branch, as CHOP and PERK mRNA levels were significantly increased after treatment with ticagrelor (Figure 6A,B). Activation of the IRE1α branch was also observed, since there was a significant increase of spliced XBP1 (XBP1-s) (Figure 6A,C), while levels of unspliced XBP1 (XBP1-u) remained unchanged (Figure 6A). The ER chaperone BiP—which can activate all three ER stress branches—showed a non-significant increase after ticagrelor treatment (Figure 6A,D) and treatment with ticagrelor did not affect the levels of ATF6 (Figure 6A). To evaluate if there was a dose-dependent effect on the expression of ER stress markers, THP1-differentiated macrophages were exposed to 0 µM, 0.5 µM and 1 µM ticagrelor. Notably, 1 µM ticagrelor lead to a 20-fold increase in the expression of CHOP (Figure 6B), which could have contributed to the toxicity of ticagrelor at higher concentrations, as CHOP is an important mediator in ER stress-induced apoptosis [30]. 

**Figure 7:**
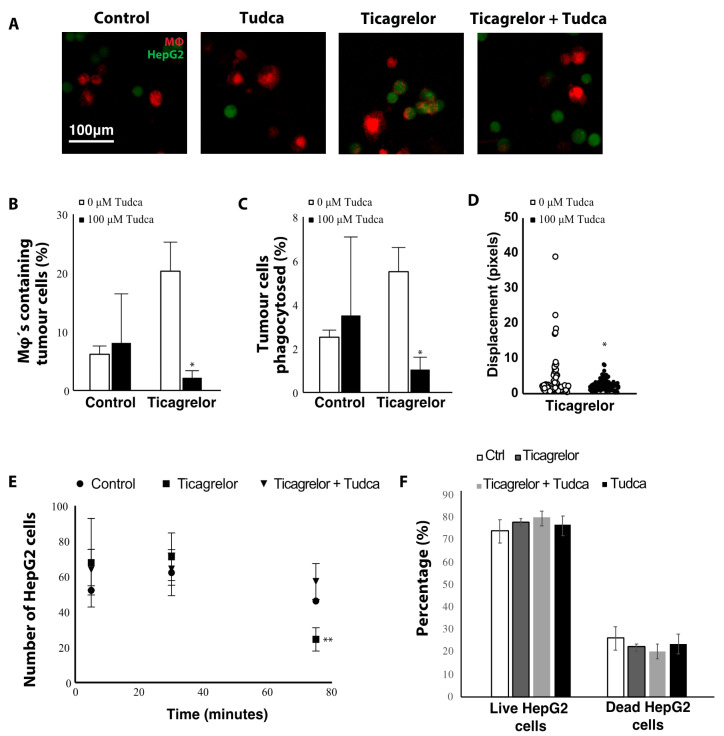
Ticagrelor affects macrophage function in an ER stress dependent manner. (**A**) Confocal microscopy images of macrophages (Mφ) and HepG2 cells stained with CellTracker Red CMTPX and Green CMFDA, respectively. Macrophages were pre-treated with 100 µM tauroursodeoxycholic acid (Tudca). HepG2 cells were added to the THP1 macrophages in combination with 0.5 µM ticagrelor and/or 100 µM Tudca. (**B**) Quantification of the percentage of macrophages that have phagocytosed tumor cells. (**C**) Quantification of the percentage of tumor cells that were removed by macrophages. (**D**) Displacement of macrophages measured on time-lapse videos of the phagocytosis assay. (**E**) Time-dependent effect on the number of HepG2 cells in the macrophage co-cultures. (**F**) Percentage of live and dead HepG2 cells in the co-cultures * = *p* < 0.05; ** = *p* < 0.01.

To assess whether inhibition of ER stress would affect phagocytosis in ticagrelor-treated macrophages, the ER stress inhibitor tauroursodeoxycholic acid (Tudca) was added to the phagocytosis assay before and during ticagrelor treatment (Figure 7A). Indeed, treatment with Tudca significantly decreased the number of macrophages phagocytosing tumor cells in the ticagrelor-treated macrophages (Figure 7B), as well as the number of tumor cells removed by macrophages (Figure 7C) and the overall movement of macrophages (Figure 7D). While a significant decrease in the number of HepG2’s was observed after 75 min of co-culture in the ticagrelor-treated group, treatment with Tudca seemed to restore this to similar levels as controls. These findings suggest that P2Y12 inhibition affects macrophage function in an ER stress dependent manner.

To assess whether the increased phagocytosis was due to a toxic effect of ticagrelor on the HepG2 cells (as macrophages would then rapidly clear the dead cells), we performed a live/dead cell assay in HepG2 cell co-cultured with THP1 macrophages. When measuring the percentage of live and dead HepG2 cells in the co-cultures, we observed no significant differences between control, ticagrelor, Tudca or the combination of ticagrelor and Tudca, suggesting that the observed effect on phagocytosis is not a result of a direct toxicity of any of the compounds used in this study (Figure 7F).

## 3. Discussion

Hepatocellular carcinoma is a clear example of an inflammation-related cancer, as more than 90% of HCC cases arise in the context of hepatic injury and inflammation. Chronic unresolved inflammation is associated with persistent hepatic injury and hepatocyte regeneration, leading to the development of fibrosis, cirrhosis, and eventually HCC. The perpetuated wound-healing response activated by parenchymal cell death and the resulting inflammatory cascade actively contributes to the hepatocarcinogenesis. Given the limited therapeutic efficacy in advanced HCC, prevention of HCC development could be an effective strategy for improving prognosis. Several pre-clinical and clinical studies have provided evidence that anti-platelet therapies, such as the P2Y12 antagonists clopidogrel and ticagrelor, slow down the progression of HCC by suppressing the intra-hepatic inflammatory response [31,32,33]. This has mainly been attributed to the effect on platelets, which drive tumorigenesis by acting on tumor cells and on different cells in the hepatic stroma [34]. In this study, we show an alternative route that can contribute to the therapeutic success of P2Y12 inhibitors. Using different animal models and cell lines, we show that P2Y12 is expressed by hepatic macrophages and provide evidence that this receptor plays a role in mediating macrophage polarization and function. 

A similar role for P2Y12 has been described in microglial cells—the resident macrophages of the central nervous system—where the P2Y12 receptor is known to be involved in chemotaxis and mediates phagocytosis in case of nerve injury [19,20,35,36]. In our study, we show for the first time that hepatic macrophages also express this purinergic receptor and that it could be involved in mediating macrophage function. In vitro treatment of macrophage cell lines with a P2Y12 antagonist increased tumor cell phagocytosis and increased the expression of the anti-tumoral macrophage marker HLA-DR, while simultaneously reducing the expression of the pro-tumoral macrophage marker arginase-1. This is in line with the study of Moore et al., which shows that P2Y12 expression is increased in alternatively activated M2 microglia and P2Y12 antagonism decreases their anti-inflammatory function [20]. P2Y12 serves as a receptor for extracellular ADP, and adenosines are known to promote an anti-inflammatory phenotype as part of the resolution phase of an inflammatory response [18]. Other studies have also shown that the macrophage phenotype and function highly depend on ADP/ATP homeostasis, and higher ADP levels are found in M2 macrophages [37]. 

In our study, we observed an increased expression of several actors of the ER stress pathway after treatment with the P2Y12 antagonist ticagrelor, and blocking the ER stress pathway with Tudca, a well-known ER stress inhibitor, diminished the ticagrelor-induced pro-phagocytotic effect. Endoplasmic reticulum stress could therefore have played a major role in mediating macrophage function. Studies have shown that activation of the ER stress pathways is associated with an increased anti-tumoral (M1) phenotype, mainly through activation of PERK [9] and CHOP [38], which were also the ER stress actors that were highest in our mRNA analyses. However, the exact mechanism on how P2Y12 inhibition induces ER stress and how this contributes to the phenotypic switch in macrophages, warrants further research. A possible explanation is that ticagrelor activates the mitogen-activated protein kinase (MAPK) pathway. Studies on gastric epithelial cells have shown that clopidogrel—another P2Y12 antagonist—induces ER stress through activation of p38-MAPK [39]. In addition, the P2Y12 receptor has been shown to act on the MAPK pathway in microglial cells during nerve injury [40]. The MAPK pathway is a critical factor in the polarization of tumor-associated macrophages, as p38-MAPK and ERK1/2 activation push M2 macrophages towards the pro-inflammatory M1 phenotype [41]. However, studies have also shown that increased expression of UPR actors, such as BiP, IRE1, spliced XBP1 and PERK in macrophages, correlates with a pro-tumoral (M2) macrophage phenotype and function [42,43,44]. For instance, targeting PERK has been shown to directly promote M1-like anti-tumoral activity and clearance of tumor cells [44]. Important to note, however, is that UPR signaling is highly dependent on the stimuli that cause ER stress, as well as the cell type and the microenvironment that are affected. This could in part explain the contradicting findings on the role of ER stress in macrophages, as different environmental cues and possibly different macrophage origins could affect the threshold at which ER stress is induced, and which UPR arms will consequently be activated. Therefore, further research is needed to explore the exact role of UPR signaling in directing macrophage polarization, function and tumor-killing capacity.

The well-characterized P2Y12 antagonist ticagrelor was used to inhibit the P2Y12 receptor. Ticagrelor inhibits ADP signaling by inducing a conformational change in P2Y12 and thereby locking the receptor in an inactive state [45]. ADP can therefore still bind at its binding site, and the degree of receptor inhibition (and inhibition of ADP-induced signaling) is dependent on the concentration of ticagrelor [46]. It has been shown that ticagrelor has a 50% inhibitory concentration on P2Y12 of 0.074 ± 0.038 μM after 1 min and reaches equilibrium within 15 min [45]. It also decreases MeSADP receptor activation at a 50% inhibitory concentration of 0.059 ± 0.03 μM [45]. In our studies, we used 0.5 μM to 1 μM ticagrelor and longer incubation times than 1 min, indicating that the concentration and time used in our studies are likely sufficient to block the P2Y12 receptor. As we have not used any additional silencing techniques, due to the low viability after nucleofection of THP1 macrophages [47], it is important to note that our observations could be caused by an off-target effect. Ticagrelor is unique in having only one well-documented additional target of inhibition, namely a weak binding to the equilibrative nucleoside transporter 1 [48]. Other studies have shown that the inhibitory effect is weak, and functional relevance is limited to concentrations higher than 10 µM [49,50], due to ticagrelor´s high affinity to the P2Y12 receptor [45]. We can indeed not exclude that this off-target effect contributed to the results in our study, especially since the equilibrative nucleoside transporter 1 mediates the cellular uptake of nucleosides from the surrounding medium. However, it is important to note that we are using a dose that is far below the previously described threshold in order to lower even more the probability of an off-target effect.

To conclude, in this study we show that P2Y12 is expressed by hepatic macrophages in mice with liver cirrhosis and hepatocellular carcinoma. In vitro inhibition of P2Y12 with ticagrelor promoted an anti-tumoral phenotype and increased the expression of several actors of the ER stress pathways. More research is needed to elucidate the mechanism behind the P2Y12-dependent increase of ER stress and how this contributed to the phenotypic switch in macrophages.

## 4. Materials and Methods

### 4.1. Animal Models

Male C57Bl/6 (Crl) or sv129 mice were obtained from Scanbur (Denmark). Mice were housed in standard conditions with a normal dark–light cycle. Animals were acclimatized to their environment for 1 week and given ad libitum access to water and food throughout the experiments. For the cirrhotic mouse model, mice were injected twice per week with CCl_4_ (Sigma) (1:1 dissolved in olive oil; 1 mg/kg), and 5% alcohol was added to drinking water [51]. For the HCC mouse model, a DEN-induced model was used as previously described [52,53]. Five-week-old male Sv129 mice received intraperitoneal injections once per week with DEN (35 mg/kg bodyweight) diluted in saline. During experiments, mice were weighted twice per week, and their health was evaluated using the Uppsala University health monitoring template. This method was approved by the local ethical committee for animal experimentation (C95/14) and conforms to the Animal Research: Reporting of In Vivo Experiments guidelines developed by the National Centre for the Replacement, Refinement and Reduction of Animals in Research.

### 4.2. Cell Culture and Reagents

The HCC cell line HepG2 was cultured in Dulbecco modified Eagle medium (DMEM) supplemented with 10% heat-inactivated fetal bovine serum (FBS) (Gibco, ThermoFisher). THP1 monocytes were cultured as suspension cells in RPMI medium. Raw264.7 cells were grown in DMEM supplemented with 10% heat-inactivated FBS (Gibco). Misidentification of the cell lines was checked at the Register of Misidentified Cell Lines, and none of the chosen cell lines were on the list. Extracted DNA from all our cell lines are sent yearly to Eurofins Genomics (Ebersberg, Germany) for cell line authentication using DNA and short tandem repeat profiles. Authentication confirmed the correct identity of each cell line.

### 4.3. Differentiation of THP1 Cells

The monocyte cell line THP-1 was differentiated to a macrophage phenotype by treatment with phorbol-12-myristate-13-acetate and then further primed towards a pro-inflammatory activation state (M1) by treatment with interferon gamma and lipopolysaccharide or an anti-inflammatory state (M2) by treatment with IL13 and IL4, as previously described [47].

### 4.4. Proliferation

Cell proliferation was monitored via a resazurin reduction assay, as previously described [54]. A 1% solution of resazurin sodium salt (Sigma-Aldrich, Darmstadt, Germany) in phosphate-buffered saline (PBS) was filtered through a 0.22 μM filter. The filtered resazurin solution was added in 1/80 dilution to the cells and incubated for 24 h, after which fluorescent signal was measured with a 540/35 excitation filter and a 590/20 emission filter on a Fluostar Omega plate reader.

### 4.5. Phagocytosis Assay

THP1-differentiated macrophages were seeded on 8-well LabTek chambers, primed to a pro-inflammatory phenotype and incubated with CellTracker Red CMTPX dye (C34552, ThermoFisher, Uppsala, Sweden). HepG2 cells were incubated with CellTracker Green CMFDA dye (C7025, ThermoFisher, Uppsala, Sweden) and added to the THP1-differentiated macrophages at a concentration of 30,000 cells per well. Simultaneously, 0.5 µM ticagrelor and/or 100 µM Tudca was added to the co-culture, followed by 30 min incubation at 37 °C to allow HepG2 cells to reach the macrophage mono-layer. In the conditions where Tudca was added, macrophages had also been pre-incubated with 100 µM Tudca, prior to adding HepG2 cells. This concentration has previously been shown to effectively inhibit ER stress in different cell types [55,56]

Cell interactions were observed and imaged with an inverted confocal microscope (LSM 700, Zeiss, Stockholm, Sweden), using Plan-Apochromat 20× objectives and the Zen 2009 software (Zeiss, Stockholm, Sweden). Quantification of the phagocytosis rate was conducted by counting the number of cells that emitted both a red and green signal using ImageJ by Fiji. The following formulas were used to determine the percentage of macrophages phagocytosing tumor cells (Equation (1)) and the percentage of tumor cells phagocytosed by macrophages (Equation (2)). 

Equation (1): Percentage of macrophages phagocytosing tumor cells

(1) $$\left[\frac{\left(Number\;of\;red\;and\;green\;cells\right)}{\left(Number\;of\;red\;and\;green\;cells\right)+\left(Number\;of\;red\;cells\right)}\right]\times 100$$

Equation (2): Percentage of tumor cells phagocytosed by macrophages

(2) $$\left[\frac{\left(Number\;of\;red\;and\;green\;cells\right)}{\left(Number\;of\;red\;and\;green\;cells\right)+\left(Number\;of\;green\;cells\right)}\right]\times 100$$

### 4.6. Immunohistochemistry

Tissue samples were fixed in 4% paraformaldehyde for 24 h and subsequently embedded in paraffin. Paraffin embedded tissue samples were cut at 5 μm and dried on SuperFrost plus glass slides overnight. Sections were de-paraffinized and rehydrated prior to staining. Antigen retrieval was performed by incubation at 37 °C with 0.05% trypsin and 0.1% CaCl_2_ solution in water. Blocking was carried out with TNB-blocking reagent (FP1020, Perkin-Elmer, Hägersten, Sweden) for 45 min and followed by an overnight incubation at 4 °C with primary antibodies against F4/80 (14-4801-82, ThermoFisher, Uppsala, Sweden) and P2Y12 (702516, ThermoFisher, Uppsala, Sweden). A 40-min incubation was used for the secondary antibody (Alexa 555 goat anti-rat (A-21434) and Alexa 488 donkey anti-rabbit (A-32790) from ThermoFisher, Uppsala, Sweden), and cell nuclei were stained with Hoechst for 8 min. 

Cells were fixed for 10 min in 4% paraformaldehyde and subsequently washed with PBS. Prior to incubation with the primary antibodies against HLA-DR (PA5-22113, ThermoFisher, Uppsala, Sweden), arginase-1 (PA5-29645, ThermoFisher, Uppsala, Sweden) and P2Y12 (702516, ThermoFisher, Uppsala, Sweden) antigens were retrieved via heat-induced incubation with citrate buffer and blocking was done with TNB-blocking reagents. A 40-min incubation was used for the secondary antibody, Alexa 488 donkey anti-rabbit, (A-32790, ThermoFisher, Uppsala, Sweden) and cell nuclei were stained with Hoechst for 8 min.

Images were taken using an inverted confocal microscope (LSM 700, Zeiss, Stockholm, Sweden) using Plan-Apochromat 20× objectives and the Zen 2009 software (Zeiss, Stockholm, Sweden). The different channels of immunofluorescent images were merged using Fiji ImageJ software. Quantifications were performed blindly with ImageJ software by conversion to binary images for each channel and automated detection of staining on thresholded images using a macro. Movement of cells was measured using the TrackMate plugin in Fiji [57] on time-lapse videos that were obtained at 30 s intervals during a 10-min period. Cell movement is expressed as displacement over their entire trajectory during 20 frames.

### 4.7. SDS-PAGE and Western Blot

Protein lysates in lysis buffer were mixed with 2× Laemmli buffer and heated to 95 °C for 5 min before being loaded onto a Precast Mini-Protean^®^ TGX™ gels (456-9034, Biorad, Solna, Sweden). After separation, proteins were transferred to an Immobilon^®^-Fl membrane (IPFL0010, Millipore, Solna, Sweden). The membrane was blocked using the Intercept^®^ (Tris Buffered Saline) blocking buffer (927-60001, Li-Cor, Bad Homburg, Germany) diluted 1:4 in PBS, and sequentially incubated with P2Y12 antibodies and goat-anti-rabbit Alexa 680 (A21088, ThermoFisher, Uppsala, Sweden) PBS + 0.1% Tween^®^20 or blocking buffer with 0.1% Tween^®^20 and 0.01% SDS, respectively. Beta-actin antibodies were used as loading control (ab6276, Abcam, Cambridge, UK) and incubated with IRDye 800CW Goat anti-mouse (Li-Cor, Bad Homburg, Germany,). All incubations were carried out at room temperature for 1 h or overnight at 4 °C. The membranes were scanned using an Odyssey scanner (Li-Cor, Bad Homburg, Germany).

### 4.8. Flow Cytometry

THP1 cells were seeded into 6-well plates at 3 × 10^5^ cells/well and differentiated to a pro-inflammatory (M1) phenotype as previously described [47]. Differentiated macrophages were then treated with 0.5 µM ticagrelor for 24 h, followed by flow cytometry to measure HLA-DR protein expression. Briefly, cells were harvested via a 15-min heat-incubation with accutase (00-4555-56, ThermoFisher, Uppsala, Sweden), pelleted, and washed with PBS. Cells were then incubated with APC anti-human HLA-DR antibody (clone L243, Biolegend, Taby, Sweden) and diluted 1:25 in cell staining buffer (420201, Biolegend, Taby, Sweden) for 45 min in the dark, at room temperature. Cells were subsequently washed three times with cell staining buffer, and fluorescence was measured using the BD Accuri C6 Plus flow cytometer. Results are shown as percentage of mean APC-HLA-DR fluorescence intensity of the HLA-DR^+^ control condition.

### 4.9. RT-qPCR

RNA was isolated from tissue or cell culture using the EZNA^®^ RNA isolation Kit II (R6934-02, VWR, Spånga, Sweden). RNA concentration and purity were assessed using Nanodrop, after which 500 ng of mRNA was reverse transcribed with an iScript select cDNA synthesis kit (1708897, Bio-Rad, Solna, Sweden). Amplifications were conducted using the following primers (Table 1). mRNA expression was normalized to 18S, GAPDH and/or TBP1. Fold change was calculated via the delta–delta CT method, by using the average CT value of 3 technical replicates.

### 4.10. Human Protein Atlas

Micrographs from biopsies from HCC patients stained with antibodies against P2Y12 were obtained through the Human Protein Atlas and are available through http://www.proteinatlas.org [25,26]. Patient survival was correlated to expression levels of P2Y12, using publicly available data from the Human Protein Atlas [25,27]. Based on the fragments per kilobase of exon per million reads (FPKM) value of each gene, patients were classified into two expression groups, and the correlation between expression level and patient survival was examined using GraphPad Prism 8. Based on the data from the Human Protein Atlas, a cut-off of 0.03 FPKM was used to determine high or low expression. This value corresponded to the best expression cut-off level, meaning the FPKM value that would yield maximum difference in regard to survival between the two groups at the lowest log-rank *p*-value. Expression of P2Y12 in different tumor stages of HCC was derived from the Human Protein Atlas [25,27] using RNA sequence data from the Cancer Genome Atlas [58].

### 4.11. Live/Dead Cell Double Staining Kit

The percentage of live and dead HepG2 cells in co-cultures was measured by using a Live/Dead Cell Double Staining Kit (04511-1KT-F, Sigma-Aldrich, Darmstadt, Germany) following the manufacturer´s guidelines. Briefly, HepG2 cells were incubated with calcein-AM and propidium iodide solutions. Stained HepG2 cells were washed and then added to the THP1-differentiated macrophages. Percentages of live and dead cells were monitored using an inverted confocal microscope (LSM 700, Zeiss, Stockholm, Sweden) using Plan-Apochromat 20× objectives and the Zen 2009 software (Zeiss, Stockholm, Sweden). As living cells emit a strong green fluorescence (λex 490 nm, λem 515 nm) and dead cells emit red fluorescence (λex 535 nm, λem 617 nm) after excitation with a 488-laser, the number of fluorescently green and fluorescently red cells was simultaneously quantified to measure the percentage of live and dead cells amongst the HepG2 population.

### 4.12. Statistics

Data are presented as mean ± standard error of the mean. Statistical significance was determined using an unpaired, two-tailed Student’s T-test, and *p*-values < 0.05 were considered statistically significant. In vitro experiments were conducted in at least 3 biological replicates, which we define as parallel measurements of biologically distinct samples. We define technical replicates as loading of the same sample multiple times on the final assay. The in vivo experiments were performed on at least 5 independent animals. 

## Figures and Tables

**Table 1:** Primer sequences used for RT-qPCR.

Target (Species)	Forward	Reverse
*P2ry12 (mouse)*	ACGAGTTTGTGAAGGCACGA	AGACTCACGACTTTCTTCTTTGGA
*P2ry12 (human)*	TTTGCCCGAATTCCTTACAC	ATTGGGGCACTTCAGCATAC
*18S (human)*	AGTCCCTGCCCTTTGTACACA	GATCCGAGGGCCTCACTAAAC
*CHOP (human)*	CATCACCACACCTGAAAGCA	TCAGCTGCCATCTCTGCA
*XBP1-spliced (human)*	AGACAGCGCTTGGGGATGGAT	CCTGCACCTGCTGCGGACTC
*BIP (human)*	GAAAGAAGGTTACCCATGCAGT	CAGGCCATAAGCAATAGCAGC
*GAPDH (human)*	GGAGCGAGATCCCTCCAAAAT	GGCTGTTGTCATACTTCTCATGG
*GAPDH (mouse)*	AATGAAGGGGTCGTTGATG	GGTGAAGGTCGGTGTGAAC
*TBP1 (human)*	AGTGACCCAGCATCACTGTTT	GGCAAACCAGAAACCCTTGC
*ATF4 (human)*	TCAGACAGTACCAACGCTTATGC	GTTGTACCACAGTAGGCTGAGA
*ATF6 (human)*	GTTGTACCACAGTAGGCTGAGA	TGGCCAAGCACTTCAAACCT
*IRE1 (human)*	TAGTCAGTTCTGCGTCCGCT	TTCCAAAAATCCCGAGGCCG
*PERK (human)*	ACGATGAGACAGAGTTGCGA	GCGCGTAAACAAGTTGCCT

## Supplementary Materials

The following are available online at https://www.mdpi.com/1422-0067/21/21/8177/s1.

## Abbreviations

ADP	Adenosine diphosphate
ATF6	Activating transcription factor 6
ATP	Adenosine triphosphate
BIP	Binding immunoglobulin protein
CCL4	Carbon tetrachloride
DEN	Diethylnitrosamine
DMEM	Dulbecco modified eagle medium
FPKM	Fragments Per Kilobase of exon per Million
HCC	Hepatocellular carcinoma
IRE1α	Inositol-requiring enzyme alpha
IL	Interleukin
MAPK	Mitogen-activated protein kinase
PBS	Phosphate buffered saline
PERK	Protein kinase R-like endoplasmic reticulum kinase
Tudca	Tauroursodeoxycholic acid
UPR	Unfolded protein response

## References

[1] Guillot A., Tacke F. (2019). Liver Macrophages: Old Dogmas and New Insights. Hepatol. Commun..

[2] Guillot A., Kohlhepp M.S., Bruneau A., Heymann F., Tacke F. (2020). Deciphering the Immune Microenvironment on a Single Archival Formalin-Fixed Paraffin-Embedded Tissue Section by An Immediately Implementable Multiplex Fluorescence Immunostaining Protocol. Cancers.

[3] Best J., Verhulst S., Syn W.K., Lagaisse K., van Hul N., Heindryckx F., Sowa J.P., Peeters L., Van Vlierberghe H., Leclercq I.A. (2016). Macrophage Depletion Attenuates Extracellular Matrix Deposition and Ductular Reaction in a Mouse Model of Chronic Cholangiopathies. PLoS ONE.

[4] Kaczmarek M., Rubis B., Frydrychowicz M., Nowicka A., Brajer-Luftmann B., Kozlowska M., Lagiedo M., Batura-Gabryel H., Sikora J. (2018). Pleural Macrophages can Promote or Inhibit Apoptosis of Malignant Cells via Humoral Mediators Depending on Intracellular Signaling Pathways. Cancer Investig..

[5] Takahashi H., Kawaguchi T., Yan L., Peng X., Qi Q., Morris L.G.T., Chan T.A., Tsung A., Otsuji E., Takabe K. (2020). Immune Cytolytic Activity for Comprehensive Understanding of Immune Landscape in Hepatocellular Carcinoma. Cancers.

[6] Chen J., Huang Z.B., Liao C.J., Hu X.W., Li S.L., Qi M., Fan X.G., Huang Y. (2020). LncRNA TP73-AS1/miR-539/MMP-8 axis modulates M2 macrophage polarization in hepatocellular carcinoma via TGF-beta1 signaling. Cell. Signal..

[7] Wu J., Gao W., Tang Q., Yu Y., You W., Wu Z., Fan Y., Zhang L., Wu C., Han G. (2020). M2 macrophage-derived exosomes facilitate hepatocarcinoma metastasis by transferring alphaM beta2 integrin to tumor cells. Hepatology.

[8] Gomez J.A., Rutkowski D.T. (2016). Experimental reconstitution of chronic ER stress in the liver reveals feedback suppression of BiP mRNA expression. Elife.

[9] Yang F., Liu Y., Ren H., Zhou G., Yuan X., Shi X. (2019). ER-stress regulates macrophage polarization through pancreatic EIF-2alpha kinase. Cell. Immunol..

[10] Zhao Y., Jiang Y., Chen L., Zheng X., Zhu J., Song X., Shi J., Li Y., He W. (2020). Inhibition of the endoplasmic reticulum (ER) stress-associated IRE-1/XBP-1 pathway alleviates acute lung injury via modulation of macrophage activation. J. Thorac. Dis..

[11] Van Campenhout S., Tilleman L., Lefere S., Vandierendonck A., Raevens S., Verhelst X., Geerts A., Van Nieuwerburgh F., Van Vlierberghe H., Devisscher L. (2020). Myeloid-specific IRE1alpha deletion reduces tumour development in a diabetic, non-alcoholic steatohepatitis-induced hepatocellular carcinoma mouse model. Metabolism.

[12] Heindryckx F., Binet F., Ponticos M., Rombouts K., Lau J., Kreuger J., Gerwins P. (2016). Endoplasmic reticulum stress enhances fibrosis through IRE1alpha-mediated degradation of miR-150 and XBP-1 splicing. EMBO Mol. Med..

[13] Borkham-Kamphorst E., Steffen B.T., van de Leur E., Haas U., Weiskirchen R. (2018). Portal myofibroblasts are sensitive to CCN-mediated endoplasmic reticulum stress-related apoptosis with potential to attenuate biliary fibrogenesis. Cell. Signal..

[14] Dasgupta D., Nakao Y., Mauer A.S., Thompson J.M., Sehrawat T.S., Liao C.Y., Krishnan A., Lucien F., Guo Q., Liu M. (2020). IRE1A Stimulates Hepatocyte-derived Extracellular Vesicles That Promote Inflammation in Mice With Steatohepatitis. Gastroenterology.

[15] Pavlović N., Calitz C., Thanapirom K., Mazza G., Rombouts K., Gerwins P., Heindryckx F. (2020). Inhibiting IRE1α-endonuclease activity decreases tumor burden in a mouse model for hepatocellular carcinoma. eLife.

[16] O’Connor S., Montalescot G., Collet J.P. (2011). The P2Y(12) receptor as a target of antithrombotic drugs. Purinergic Signal..

[17] Mansour A., Bachelot-Loza C., Nesseler N., Gaussem P., Gouin-Thibault I. (2020). P2Y12 Inhibition beyond Thrombosis: Effects on Inflammation. Int. J. Mol. Sci..

[18] Zanin R.F., Braganhol E., Bergamin L.S., Campesato L.F., Filho A.Z., Moreira J.C., Morrone F.B., Sevigny J., Schetinger M.R., de Souza Wyse A.T. (2012). Differential macrophage activation alters the expression profile of NTPDase and ecto-5’-nucleotidase. PLoS ONE.

[19] Haynes S.E., Hollopeter G., Yang G., Kurpius D., Dailey M.E., Gan W.B., Julius D. (2006). The P2Y12 receptor regulates microglial activation by extracellular nucleotides. Nat. Neurosci..

[20] Moore C.S., Ase A.R., Kinsara A., Rao V.T., Michell-Robinson M., Leong S.Y., Butovsky O., Ludwin S.K., Seguela P., Bar-Or A. (2015). P2Y12 expression and function in alternatively activated human microglia. Neurol. Neuroimmunol. Neuroinflamm..

[21] Schrottmaier W.C., Kral J.B., Badrnya S., Assinger A. (2015). Aspirin and P2Y12 Inhibitors in platelet-mediated activation of neutrophils and monocytes. Thromb. Haemost..

[22] Van Kolen K., Gilany K., Moens L., Esmans E.L., Slegers H. (2006). P2Y12 receptor signalling towards PKB proceeds through IGF-I receptor cross-talk and requires activation of Src, Pyk2 and Rap1. Cell. Signal..

[23] Li F., Xu D., Hou K., Gou X., Li Y. (2020). The role of P2Y12 receptor inhibition in ischemic stroke on microglia, platelets and vascular smooth muscle cells. J. Thromb. Thrombolysis.

[24] Rauch B.H., Rosenkranz A.C., Ermler S., Bohm A., Driessen J., Fischer J.W., Sugidachi A., Jakubowski J.A., Schror K. (2010). Regulation of functionally active P2Y12 ADP receptors by thrombin in human smooth muscle cells and the presence of P2Y12 in carotid artery lesions. Arterioscler. Thromb. Vasc. Biol..

[25] Uhlen M., Fagerberg L., Hallstrom B.M., Lindskog C., Oksvold P., Mardinoglu A., Sivertsson A., Kampf C., Sjostedt E., Asplund A. (2015). Proteomics. Tissue-based map of the human proteome. Science.

[26] Proteinatlas Images. https://www.proteinatlas.org/ENSG00000169313-P2RY12/pathology/liver+cancer#img.

[27] Proteinatlas Data. https://www.proteinatlas.org/ENSG00000169313-P2RY12/pathology/liver+cancer.

[28] Mira-Pascual L., Tran A.N., Andersson G., Nareoja T., Lang P. (2020). A Sub-Clone of RAW264.7-Cells Form Osteoclast-Like Cells Capable of Bone Resorption Faster than Parental RAW264.7 through Increased De Novo Expression and Nuclear Translocation of NFATc1. Int. J. Mol. Sci..

[29] Chanput W., Mes J.J., Wichers H.J. (2014). THP-1 cell line: An in vitro cell model for immune modulation approach. Int. Immunopharmacol..

[30] Hu H., Tian M., Ding C., Yu S. (2018). The C/EBP Homologous Protein (CHOP) Transcription Factor Functions in Endoplasmic Reticulum Stress-Induced Apoptosis and Microbial Infection. Front. Immunol..

[31] Malehmir M., Pfister D., Gallage S., Szydlowska M., Inverso D., Kotsiliti E., Leone V., Peiseler M., Surewaard B.G.J., Rath D. (2019). Platelet GPIbalpha is a mediator and potential interventional target for NASH and subsequent liver cancer. Nat. Med..

[32] Sitia G., Iannacone M., Guidotti L.G. (2013). Anti-platelet therapy in the prevention of hepatitis B virus-associated hepatocellular carcinoma. J. Hepatol..

[33] Tan-Shalaby J.L. (2014). Reproducible complete remission of advanced hepatocellular carcinoma with sorafenib in combination with clopidogrel. BMJ Case Rep..

[34] Pavlovic N., Rani B., Gerwins P., Heindryckx F. (2019). Platelets as Key Factors in Hepatocellular Carcinoma. Cancers.

[35] Amadio S., Parisi C., Montilli C., Carrubba A.S., Apolloni S., Volonte C. (2014). P2Y(12) receptor on the verge of a neuroinflammatory breakdown. Mediat. Inflamm..

[36] Irino Y., Nakamura Y., Inoue K., Kohsaka S., Ohsawa K. (2008). Akt activation is involved in P2Y12 receptor-mediated chemotaxis of microglia. J. Neurosci. Res..

[37] Chen W., Sandoval H., Kubiak J.Z., Li X.C., Ghobrial R.M., Kloc M. (2018). The phenotype of peritoneal mouse macrophages depends on the mitochondria and ATP/ADP homeostasis. Cell. Immunol..

[38] Suzuki T., Gao J., Ishigaki Y., Kondo K., Sawada S., Izumi T., Uno K., Kaneko K., Tsukita S., Takahashi K. (2017). ER Stress Protein CHOP Mediates Insulin Resistance by Modulating Adipose Tissue Macrophage Polarity. Cell Rep..

[39] Wu H.L., Duan Z.T., Jiang Z.D., Cao W.J., Wang Z.B., Hu K.W., Gao X., Wang S.K., He B.S., Zhang Z.Y. (2013). Increased endoplasmic reticulum stress response is involved in clopidogrel-induced apoptosis of gastric epithelial cells. PLoS ONE.

[40] Kobayashi K., Yamanaka H., Fukuoka T., Dai Y., Obata K., Noguchi K. (2008). P2Y12 receptor upregulation in activated microglia is a gateway of p38 signaling and neuropathic pain. J. Neurosci..

[41] Chakraborty P., Chatterjee S., Ganguly A., Saha P., Adhikary A., Das T., Chatterjee M., Choudhuri S.K. (2012). Reprogramming of TAM toward proimmunogenic type through regulation of MAP kinases using a redox-active copper chelate. J. Leukoc. Biol..

[42] Cubillos-Ruiz J.R., Mohamed E., Rodriguez P.C. (2017). Unfolding anti-tumor immunity: ER stress responses sculpt tolerogenic myeloid cells in cancer. J. Immunother. Cancer.

[43] Oh J., Riek A.E., Weng S., Petty M., Kim D., Colonna M., Cella M., Bernal-Mizrachi C. (2012). Endoplasmic reticulum stress controls M2 macrophage differentiation and foam cell formation. J. Biol. Chem..

[44] Soto-Pantoja D.R., Wilson A.S., Clear K.Y., Westwood B., Triozzi P.L., Cook K.L. (2017). Unfolded protein response signaling impacts macrophage polarity to modulate breast cancer cell clearance and melanoma immune checkpoint therapy responsiveness. Oncotarget.

[45] Husted S., van Giezen J.J. (2009). Ticagrelor: The first reversibly binding oral P2Y12 receptor antagonist. Cardiovasc. Ther..

[46] Hoffmann K., Lutz D.A., Strassburger J., Baqi Y., Muller C.E., von Kugelgen I. (2014). Competitive mode and site of interaction of ticagrelor at the human platelet P2Y12 -receptor. J. Thromb. Haemost..

[47] Maess M.B., Wittig B., Lorkowski S. (2014). Highly efficient transfection of human THP-1 macrophages by nucleofection. J. Vis. Exp..

[48] Sumaya W., Storey R.F. (2017). Ticagrelor: Effects Beyond the P2Y12 Receptor. Interv. Cardiol. Clin..

[49] Armstrong D., Summers C., Ewart L., Nylander S., Sidaway J.E., van Giezen J.J. (2014). Characterization of the adenosine pharmacology of ticagrelor reveals therapeutically relevant inhibition of equilibrative nucleoside transporter 1. J. Cardiovasc. Pharmacol. Ther..

[50] Aungraheeta R., Conibear A., Butler M., Kelly E., Nylander S., Mumford A., Mundell S.J. (2016). Inverse agonism at the P2Y12 receptor and ENT1 transporter blockade contribute to platelet inhibition by ticagrelor. Blood.

[51] Scholten D., Trebicka J., Liedtke C., Weiskirchen R. (2015). The carbon tetrachloride model in mice. Lab. Anim..

[52] Heindryckx F., Bogaerts E., Coulon S.H., Devlies H., Geerts A.M., Libbrecht L., Stassen J.M., Carmeliet P., Colle I.O., Van Vlierberghe H.R. (2012). Inhibition of the placental growth factor decreases burden of cholangiocarcinoma and hepatocellular carcinoma in a transgenic mouse model. Eur. J. Gastroenterol. Hepatol..

[53] Heindryckx F., Mertens K., Charette N., Vandeghinste B., Casteleyn C., Van Steenkiste C., Slaets D., Libbrecht L., Staelens S., Starkel P. (2010). Kinetics of angiogenic changes in a new mouse model for hepatocellular carcinoma. Mol. Cancer.

[54] Calitz C., Pavlović N., Rosenquist J., Zagami C., Samanta A., Heindryckx F. (2020). A Biomimetic Model for Liver Cancer to Study Tumor-Stroma Interactions in a 3D Environment with Tunable Bio-Physical Properties. J. Vis. Exp..

[55] Lin R.C., Yang S.F., Chiou H.L., Hsieh S.C., Wen S.H., Lu K.H., Hsieh Y.H. (2019). Licochalcone A-Induced Apoptosis Through the Activation of p38MAPK Pathway Mediated Mitochondrial Pathways of Apoptosis in Human Osteosarcoma Cells In Vitro and In Vivo. Cells.

[56] Park Y.R., Park H.B., Kim M.J., Jung B.D., Lee S., Park C.K., Cheong H.T. (2019). Effects of Endoplasmic Reticulum Stress Inhibitor Treatment during the Micromanipulation of Somatic Cell Nuclear Transfer in Porcine Oocytes. Dev. Reprod..

[57] Tinevez J.Y., Perry N., Schindelin J., Hoopes G.M., Reynolds G.D., Laplantine E., Bednarek S.Y., Shorte S.L., Eliceiri K.W. (2017). TrackMate: An open and extensible platform for single-particle tracking. Methods.

[58] Wheeler D.A., Roberts L.R., Cancer Genome Atlas Research Network (2017). Comprehensive and Integrative Genomic Characterization of Hepatocellular Carcinoma. Cell.

