# Supplementary figures and images for: Inhibiting P2Y12 in Macrophages Induces Endoplasmic Reticulum Stress and Promotes an Anti-Tumoral Phenotype
Source: Int J Mol Sci. 2020 Oct 31;21(21):8177. doi: 10.3390/ijms21218177 (PMC7672568; doi:10.3390/ijms21218177)

Figure 1E

Kaleidoscope Ladder

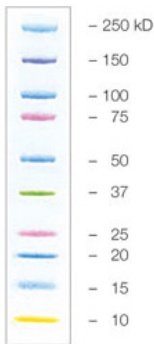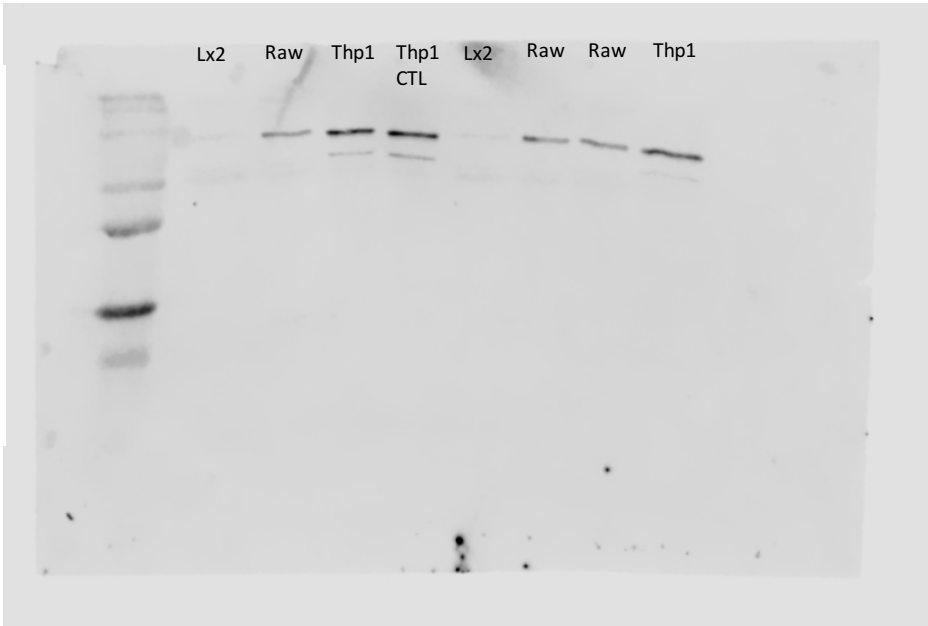

Figure 1H

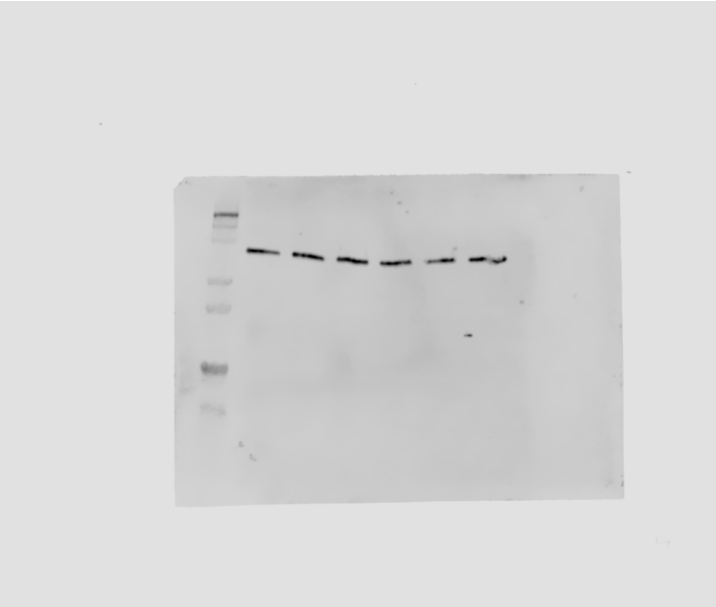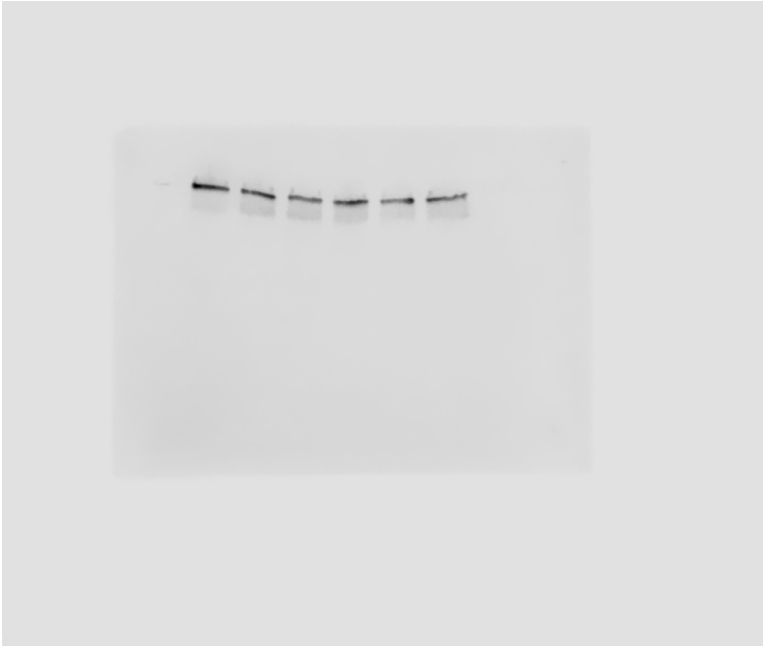

Supplement: Supplementary file 1 [file ijms-21-08177-s001.zip › Uncut WB.pdf]
